# A randomized, double-blind, placebo-controlled, cross-over pilot study to investigate the efficacy of Rest-ZZZ formula in healthy participants with occasional sleeplessness

**DOI:** 10.1007/s41105-022-00416-2

**Published:** 2022-08-22

**Authors:** Marc Moulin, Erin D. Lewis, David C. Crowley, Jamie Langston, Malkanthi Evans

**Affiliations:** 1 KGK Science Inc, London, ON Canada; 2 LifeSeasons, Inc., Lewisville, TX USA

**Keywords:** Sleep, Rest-ZZZ, Sleep quality, Diphenhydramine, Sleep efficiency

## Abstract

The purpose of this study was to investigate the safety and efficacy of Rest-ZZZ, a natural sleep supplement, in healthy adults without a diagnosed sleep disorder. This randomized, double-blind, placebo-controlled, cross-over study consisted of three 7-day supplementation periods with either Rest-ZZZ, Diphenhydramine (DPH), or Placebo, with a 7-day washout. Twenty-seven participants were randomized to one of three intervention sequences and the Healthy People Sleep Quality Index (HPSQI), Quality of Life (QoL), and Profile of Mood States (POMS) questionnaires were assessed at the beginning and end of each supplementation period. Rest-ZZZ and Placebo showed improvements in sleep-related QoL (*p* ≤ 0.044) and total mood disturbance (TMD) (*p* =  ≤ 0.028). Fatigue–Inertia was reduced by all study products (*p* ≤ 0.031). DPH did not result in any significant improvements on sleep quality parameters. A subgroup analysis of participants ≤ 45 years found enhanced efficacy of Rest-ZZZ and improvement in sleep-related QoL vs. Placebo (*p* = 0.007), as well as improvements in sleep duration (*p* = 0.007), sleep debt (*p* = 0.011), and sleep-related QoL (*p* = 0.033). DPH supplementation resulted in significant improvement in only sleep debt (*p* = 0.038). Rest-ZZZ had a safe hematology and chemistry profile. Rest-ZZZ resulted in restful sleep that generated corresponding improvements in sleep-related QoL and overall mood. Rest-ZZZ is an efficacious sleep supplement with a favorable safety profile, particularly in those aged 25–45 years.

## Introduction

Less than 40% of North American adults between the ages of 40–64 take sleep into account when planning their next day [1]. 25–33% of the population report having difficulty falling asleep and/or staying asleep [2] and 34% report a sleep disturbance in the last 7 days [3]. Data suggest that healthy people without a diagnosis of a chronic sleep disorder have interrupted sleep due to the fast pace and demand of twenty-first century living. Pharmacological sleep therapy are employed by 4% of US adults [4]. However, such therapies are associated with serious long-term adverse gastrointestinal and neurological effects [4]. Over 21% of older adults in the US reported using over-the-counter (OTC) sleep aids [5], such as Diphenhydramine (DPH), a common ingredient in night-time cold medications that induces a sedative effect by competitively blocking histamine binding [6]. Only short-term use of DPH is recommended when used for sleep as tolerance to its hypnotic effects have been reported after only 4 days of administration [7]. The anticholinergic properties of DPH result in mild to moderate side effects that may progress to severe [6]. Adverse events and concern for misuse highlight the need for safe and effective alternatives for sleep management. Rest-ZZZ (LifeSeasons, Inc.) is a natural sleep supplement containing active ingredients independently shown to improve various sleep quality (SQ) parameters [8–14]. Making up the largest quantity of active ingredients in Rest-ZZZ, melatonin promotes sleep as a result of the activation of high-affinity, G protein coupled receptors, MT1 and MT2 [15]. MT1 is mainly associated with the regulation of rapid eye movement (REM) sleep, while MT2 regulates non-REM sleep [15]. However, the synergistic effect of melatonin with valerian root extract, a herbal remedy that induces sleep through the gamma-aminobutyric acid (GABA) receptor system [16], and other natural sleep ingredients has yet to be explored.

The objective of this study was to investigate the safety and efficacy of Rest-ZZZ in healthy adults with occasional sleeplessness.

## Materials and methods

### Study design

The study was conducted at KGK Science Inc., London, Canada from September 2019 to December 2019. All participants provided written informed consent prior to any study procedures.

Participants met the following inclusion criteria: occasional sleeplessness, defined as: difficulty falling asleep (taking longer than 30 min to fall asleep) or staying asleep, with 2 or more waking episodes in a 7-day period for at least 2 weeks); Participants were required to: maintain their current sleep schedule and avoid caffeine after 3:00 p.m.; refrain from and wash-out of any OTC sleep aids; and complete questionnaires and diaries associated with the study.

Individuals were excluded for the following: previous diagnosis of a sleep disorder or used continuous positive air pressure; unstable chronic conditions that consistently interfered with sleep; conditions that may adversely affect participant ability to complete the study, or which posed a significant health risk.

**Fig. 1:**
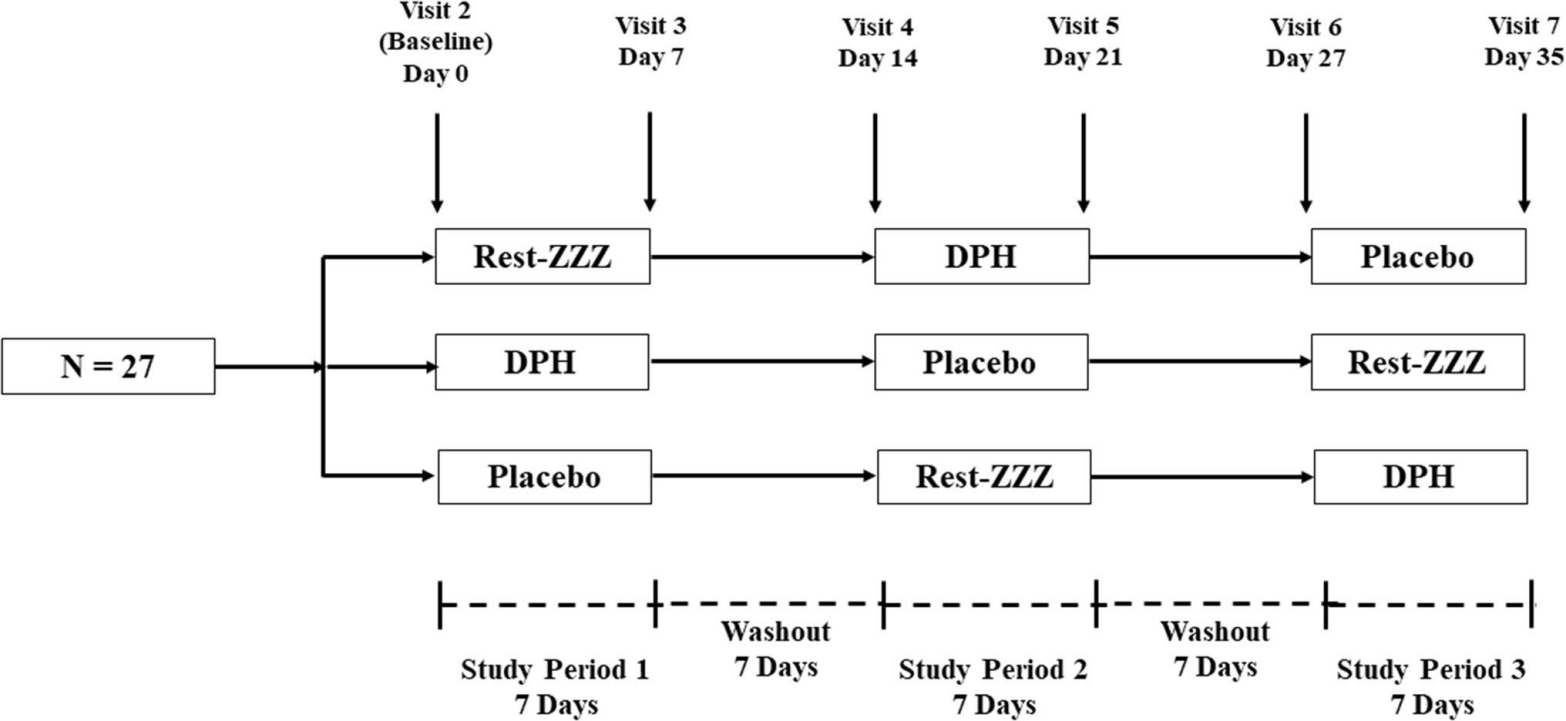
Study design demonstrating supplementation and washout periods

The study consisted of three 7-day supplementation periods with Rest-ZZZ, DPH, or Placebo. Each supplementation period was separated by a 7-day washout period (Fig. 1).

Clinical assessments for anthropometrics and vital signs were completed at every clinic visit and blood draws for complete blood count (CBC) and liver and kidney function were completed at screening and at the end of each 7-day period. A daily diary recorded concomitant therapies, investigational product (IP) use, and adverse events (AEs). Diaries were reviewed by study coordinators at each clinic visit to assess AEs and compliance. The Healthy People Sleep Quality Index (HPSQI), Quality of Life, and Profile of Mood States (POMS) questionnaires were completed at the beginning and end of each study period.

### Investigational product

During each 7-day supplementation period, participants were provided with two bottles of test product and instructed to take one capsule from each bottle 20–30 min before bed (Table 1).

**Table 1:** Active ingredients dispensed during each supplementation period (per dose)

Study product	Capsule 1	Capsule 2	Active ingredients	Non-active ingredients
Rest-ZZZ	Rest-ZZZ	Rest-ZZZ	1 mg Melatonin 300 mg Valerian root extract 200 mg German chamomile extract 175 mg Passion flower extract 100 mg GABA 100 mg Hawthorn berry 50 mg Lemon balm leaf	Vegetable cellulose, maltodextrin, rice bran, Hypromellose, titanium dioxide, sodium copper chlorophyllin, and silica
Comparator	DPH	Placebo	25 mg Diphenhydramine HCL	Vegetable cellulose, rice bran, hypromellose, titanium dioxide, sodium copper chlorophyllin, croscarmellose sodium, dicalcium phosphate, lactose, magnesium stearate, microcrystalline cellulose, mineral oil, silica, stearic acid, talc, titanium dioxide, triacetin
Placebo	Placebo	Placebo	None	Vegetable cellulose, rice bran, Hypromellose, titanium dioxide, and sodium copper chlorophyllin

### Outcomes

#### Sleep quality

The primary outcome measure was SQ and was assessed by the HPSQI. The HPSQI was designed to measure expected changes with nutraceutical supplementation in non-pathological populations and has been successfully used to assess SQ in > 260 healthy participants. Other sleep questionnaires, such as the Pittsburgh Sleep Quality Index (PSQI), are often used as the first diagnostic test in determining sleep-related pathologies [17, 18]. Similar to the PSQI, the HPSQI captures information related to the time participants went to sleep and how much sleep they think they got, and then assesses components of self-perceived SQ. However, the HPSQI assesses sleep-related quality of life (QoL) using seven questions assessed on a 5-points Likert scale to provide a comprehensive measure of daytime functioning, while the PSQI uses only two. Previous research has questioned the validity of the PSQI daytime dysfunction subscale and noted that it did not provide a clear distinction between sleep problems and daytime functioning [19, 20].

#### Quality of life

The Quality of Life Questionnaire measured discomfort and well-being. Participants answered 31 questions with a score from 1 to 7, with 1 meaning ‘Never’, 4 meaning ‘Sometimes’, and 7 meaning ‘Always’. The Quality of Life Questionnaire provided an overall measure of QoL and was not specific to sleep.

#### Profile of mood states

The POMS Questionnaire is an assessment of mood that is adaptable to capturing transient and fluctuating feelings, or relatively enduring affect states and contributes to a comprehensive assessment by providing indications of potential mood disturbance [21]. The questionnaire consisted of a list of 65 words describing feelings participants experienced after consuming the IP for the 7-day supplementation period. Items were scored on a 5-points Likert scale where 0 represented ‘Not at All’ and 4 represented ‘Extremely.’

#### Safety

Blood drawn at screening, and at days 7, 21, and 35 was analyzed by Dynacare (London, Canada). The tests included CBC and liver and kidney function. Urine pregnancy tests were conducted at the KGK clinic at screening and baseline (females only).

Participants recorded AEs in their daily diary which were classified by the Medical Director using the MEDRA 22.1 coding.

### Statistical analysis

Twenty-seven participants were enrolled in this study and accounted for 20% attrition rate and aligned with guidelines for pilot studies [22]. Analyses were conducted for intention-to-treat (ITT) and per protocol (PP) populations. The PP population consisted of all participants who consumed at least 80% of study product doses, did not have any major protocol violations, and completed all study visits and procedures related to the measurement of the primary outcome.

SQ was evaluated using the following variables: sleep efficiency (> 85% considered normal), satisfaction with SQ, sleep duration, sleep latency (time taken to fall asleep), sleep debt or sleep loss (calculated as: preferred sleep duration minus estimated sleep duration), and sleep-related QoL. Secondary outcomes included (1) the change in QoL assessed by the Quality of Life Questionnaire and (2) the change in POMS questionnaire scores from pre-supplementation to day 7 of supplementation. Safety was assessed by continuous safety variables vital signs, anthropometrics, clinical and hematological parameters, and AEs.

Outcomes were assessed for possible differences at post-supplementation between the study groups by using Mixed Model ANCOVA. Each model included study group, sequence and period as fixed effects, subject as random effect and pre-supplementation values of the dependent variable as covariate. Between group *p* values were obtained from this model, while within group *p* values were obtained using paired *t* tests or Wilcoxon Signed Rank tests as appropriate. Analysis on the effects of age (> 45 and ≤ 45 years) was explored by examining the respective subgroups. The subgroup analyses were conducted using the same methods described above. Probabilities ≤ 0.05 were considered statistically significant. All statistical analyses were completed using R version 3.5.3 [23], RStudio version 1.2.1335 [24] for Microsoft Windows, nlme package (for mixed models) [25] and related packages.

## Results

### Study participant dispositions

**Fig. 2:**
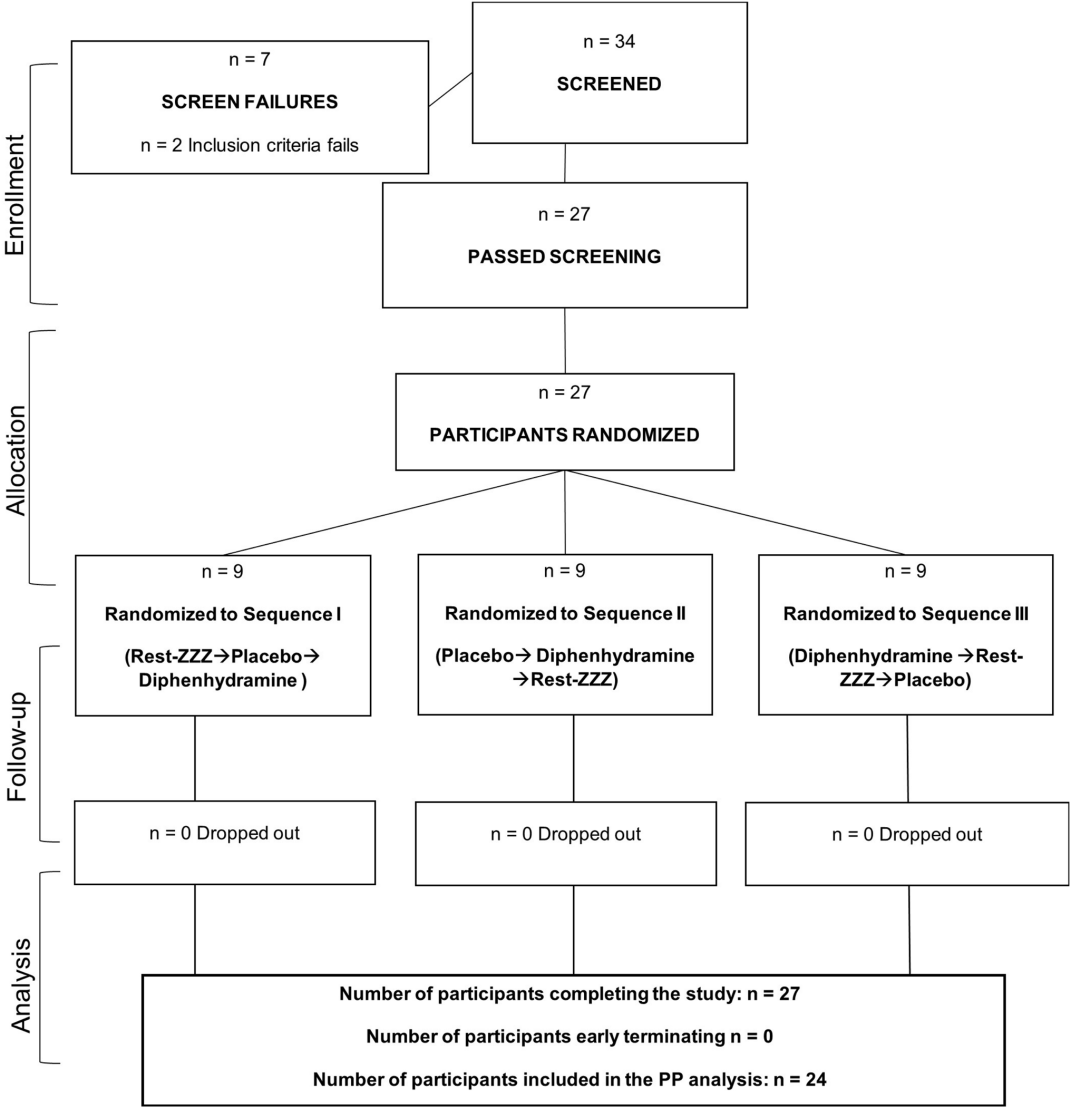
Disposition of study participants

Thirty-four participants screened, and 27 randomized to one of three sequences, with nine participants per sequence. Following a review of participant compliance and protocol deviations, a total of 24 participants were included in the PP population. Three participants were excluded from the PP population due to repeated instances of non-compliance (Fig. 2).

Participant demographics are given in Table 2. All participants were deemed healthy based on their anthropometric parameters, vital signs, electrolytes, and hematology and clinical chemistry measurements.

**Table 2:** Baseline characteristics for all enrolled participants (*n* = 27)

Demographic characteristics	
Characteristics	Mean ± SD
Age (years)	46 ± 11.92
Gender (*n*)	
Female	19
Male	8
Systolic blood pressure (mmHg)	116.86 ± 10.29
Diastolic blood pressure (mmHg)	72.07 ± 8.29
Heart rate (bpm)	69.04 ± 10.49
Weight (kg)	70.1 ± 11.6
BMI (kg/m^2^)	25.4 ± 2.96
Ethnicity [*n* (%)]	
East Asian	1 (3.7%)
Native American	1 (3.7%)
African American	1 (3.7%)
Hispanic or Latino	1 (3.7%)
South American	2 (7.4%)
Eastern European White	2 (7.4%)
Western European White	19 (70.4%)

*n* number, *SD* standard deviation

### Sleep quality

Changes in SQ parameters between study products can be found in Table 3. There were no significant differences in the change of scores for sleep efficiency, sleep duration, sleep latency, or sleep debt at Day 7.

**Table 3:** Change in sleep quality parameters from pre-supplementation to Day 7 in the PP population (*n* = 24)

	Rest-ZZZ Mean ± SD Within Group *p* value^†^ Pairwise *p* value^‡^	Placebo Mean ± SD Within Group *p* Value^†^ Pairwise *p* value^‡^	Diphenhydramine Mean ± SD Within Group *p* value^†^ Pairwise *p* value^‡^
Sleep efficiency (%)			
Change from pre-supplementation to Day 7	4.85 ± 16.19	5.57 ± 18.16	2.59 ± 18.33
	0.156	0.146	0.375 (w)
	0.180	0.978	0.174
Satisfaction with sleep quality			
Change from pre-supplementation to Day 7	− 0.50 ± 0.98	− 0.42 ± 0.97	− 0.46 ± 1.38
	0.024 (w)*	0.067 (w)	0.093 (w)
	0.658	0.654	0.382
Sleep duration (h)			
Change from pre-supplementation to Day 7	0.49 ± 1.36	0.10 ± 1.43	0.61 ± 1.46
	0.090	0.746	0.053
	0.984	0.819	0.839
Sleep latency (h)			
Change from pre-supplementation to Day 7	− 0.36 ± 1.24	− 0.58 ± 1.73	− 0.31 ± 1.61
	0.165	0.299	0.604
	0.226	0.896	0.281
Sleep debt (h)			
Change from pre-supplementation to Day 7	− 0.35 ± 1.39	0.04 ± 1.73	− 0.57 ± 1.42
	0.237	0.807 (w)	0.060
	0.790	0.805	0.981
Sleep-related quality of life			
Change from pre-supplementation to Day 7	1.88 ± 3.07	1.42 ± 3.26	1.17 ± 4.17
	0.006*	0.044*	0.183
	0.433	0.423	0.993

*SD* standard deviation, *Min* minimum, *Max* maximum

^*^Significant difference (*p* ≤ 0.05)

^**†**^Within group *p* values were generated by the Paired *t* test or the Wilcoxon Signed Rank test, indicated by (*w*)

^‡^Pairwise between group *p* values were obtained from the Mixed Model without adjustment. Pairwise between group *p* values are Rest-ZZZ versus Placebo in the first column, Rest-ZZZ versus Diphenhydramine in the second column, and Placebo versus Diphenhydramine in the last column

**Fig. 3:**
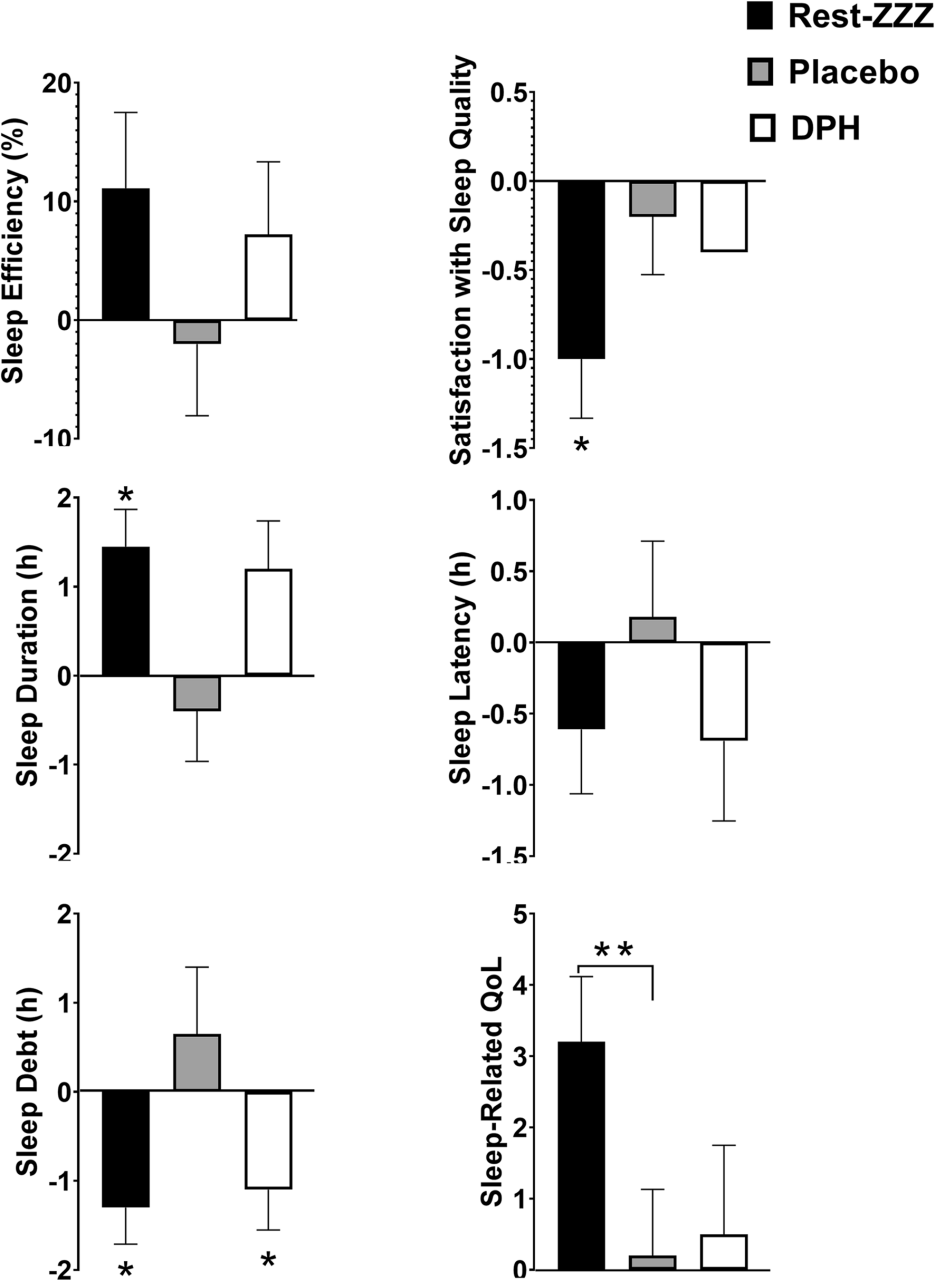
Change in SQ parameters from pre-supplementation to Day 7 in the ≤ 45 years of age population (*n* = 10). All outcomes were assessed using Mixed Model ANCOVA which included study group, sequence and period as fixed effects, subject as random effect and pre-supplementation values of the dependent variable as covariate. Between group *p* values were obtained from this model. Within group *p* values were obtained using paired *t* tests or Wilcoxon Signed Rank tests as appropriate. *p* ≤ 0.05 considered statistically significant for **between group differences and *within group differences

The proportion of participants who reported normal sleep efficiency following a7-day period with Rest-ZZZ was 12.5%. DPH showed greater improvement compared to Placebo (8.3% vs. 4.2%). Rest-ZZZ increased sleep duration by a total of 29 min (*p* = 0.09), while sleep duration with Placebo increased by only 6 min. DPH increased sleep duration by a total of 36 min (*p* = 0.053). Participants taking Rest-ZZZ (*p* = 0.006) and Placebo (*p* = 0.044) reported improvements in sleep-related QoL that corresponded with reductions in satisfaction with SQ (*p* =  ≥ 0.067). Participants taking DPH (*p* = 0.093) also reported reductions in satisfaction with SQ. SQ parameters did not show significant changes from baseline after 7 days in participants taking DPH. A subgroup analysis of those ≤ 45 years revealed significant differences across multiple SQ parameters (Fig. 3).

Participants ≤ 45 years reported significant improvements in sleep-related QoL with Rest-ZZZ vs. Placebo (*p* = 0.033). In addition, sleep duration (*p* = 0.007), sleep debt (*p* = 0.011), and sleep-related QoL (*p* = 0.007) improved from baseline after 7 days of supplementation with Rest-ZZZ. Similar to the results of the PP population, the subgroup showed that supplementation with Rest-ZZZ (*p* = 0.034) was associated with reductions in satisfaction with SQ. In participants ≥ 45 years, significant changes from baseline were limited to the Placebo, and included improvements in sleep efficiency (*p* = 0.023), sleep latency (*p* = 0.019), and sleep-related QoL (*p* = 0.022).

### Quality of life

There were no significant between-group differences in total QoL scores from pre-supplementation to Day 7. There was a significant improvement in total QoL score for participants taking Placebo (6.0 ± 13.59; *p* = 0.041), but not for Rest-ZZZ (4.38 ± 13.15; *p* = 0.117). DPH was not associated with significant improvement in total QoL (4.12 ± 12.56; *p* = 0.121).

### Profile of mood states

**Fig. 4:**
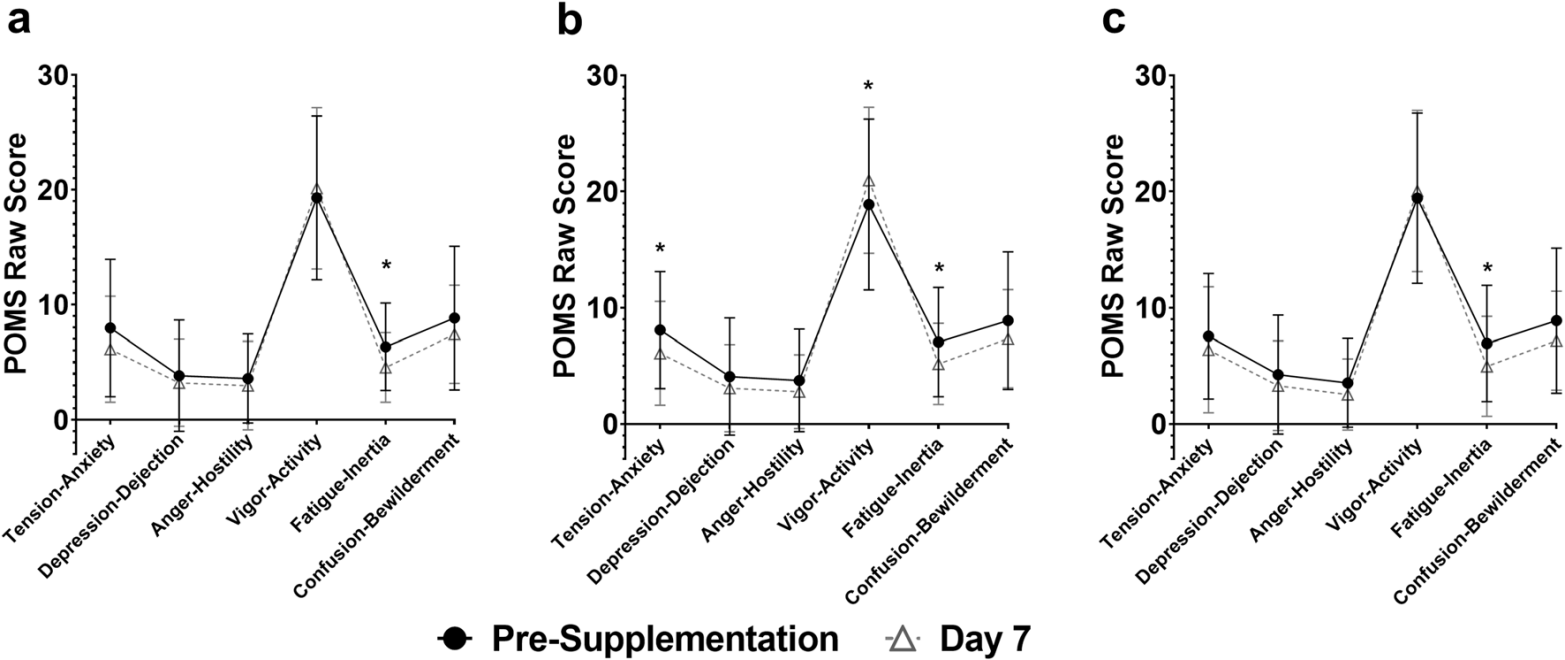
Profile of Mood States (POMS) iceberg profiles for **a** Rest-ZZZ, **b** Placebo, and **c** DPH from pre-supplementation to Day 7 in the PP population (*n* = 24). *Indicates a statistically significant difference between pre-supplementation and Day 7

There were no significant between-group differences in the change in mood states from pre- to post-supplementation at Day 7. Significant improvements in Total Mood Disturbance (TMD) scores were reported both by participants taking Rest-ZZZ (− 7.12 ± 14.84; *p* = 0.028) or Placebo (− 9.46 ± 19.52; *p* = 0.026). Participants taking DPH did not show significant improvement in TMD (− 7.42 ± 20.19). Fatigue–Inertia was significantly decreased by 28.3% with Rest-ZZZ (*p* = 0.008), compared to 26.7% with Placebo (*p* = 0.026).Participants taking DPH had a 28.3% reduction in Fatigue–Inertia (*p* = 0.031). All study products led to non-significant reductions in Anger–Hostility, Confusion–Bewilderment, and Depression–Dejection (Fig. 4).

### Safety

There were no clinically relevant changes in vital signs, hematology, kidney/liver markers, or electrolytes from pre-supplementation at Day 7. Fifteen AEs were reported by eight participants, of which three were reported while participants were supplementing with Rest-ZZZ. Six AEs were reported on DPH and Placebo. All events were classified as “not related” or “unlikely related” except for one AE, reported by a participant on Placebo as “trouble sleeping” and classified as “possibly” related to the study product.

## Discussion

Consistent with literature demonstrating a large placebo effect (39–100%) in pharmacological sleep studies [26], a 77.3% placebo effect was found for sleep-related QoL in this study. However, the Placebo was rarely associated with any significant sleep-related changes. Rest-ZZZ outperformed Placebo in all SQ parameters except sleep efficiency and satisfaction with SQ. Individual participant data revealed that the proportion of participants who achieved normal sleep efficiency while supplementing with Rest-ZZZ was greater than that of Placebo by 8.3%.

Rest-ZZZ supplementation resulted in improvements in sleep duration, efficiency, latency, and debt that corresponded with significant enhancement of sleep-related QoL, a comprehensive measure of daytime functioning as influenced by sleep. The sleep-related QoL questions within the HPSQI assessed sleepiness upon waking and throughout the morning and afternoon, as well as concentration, mood, and one’s ability to complete day-to-day activities. The results suggest that Rest-ZZZ is associated with restful sleep that leads to an improved QoL when awake. Previous sleep research has demonstrated that shorter sleep duration with better SQ is more beneficial for brain and heart health [27, 28], suggesting that the more restful sleep provided by Rest-ZZZ may be associated with better health outcomes.

A review of individual participant data confirmed high inter- and intra-individual variability across all SQ parameters. This variability may be explained by the small sample size and lifestyle factors of participants. To build upon this study and reduce the variability among participants, larger studies on the effect of Rest-ZZZ should be conducted in the future. Researchers should also consider restricting the alcohol intake of participants as previous literature has demonstrated an association between alcohol use and poor sleep [29], particularly among women [30]. The alcohol consumption of participants in this study, of which 70% were women, may have contributed to poorer sleep and blunted the effect of Rest-ZZZ. Furthermore, the healthy status of participants may have contributed to variability as healthy people are more likely to experience transient disturbances in SQ compared to a population diagnosed with a sleep disorder.

The analysis of subgroups revealed similar, yet enhanced efficacy of Rest-ZZZ to improve SQ in participants ≤ 45 years. Importantly, Rest-ZZZ resulted in a significant improvement in sleep-related QoL compared to Placebo, emphasizing the efficacy of Rest-ZZZ in this younger population. Previous research demonstrated that those < 40 years were more sensitive to the sleep-improving effects of valerian extract [31], an active ingredient in Rest-ZZZ. This may be explained by the fact that sleep changes as humans age, with older adults experiencing greater sleep difficulties compared to the younger population [32]. It is possible that the effect of Rest-ZZZ was diminished in the older age-group due to age associated challenges. Due to increased sleep difficulty and additional life stressors experienced by aging adults, a longer Rest-ZZZ supplementation period may be needed. Future investigations should explore whether adults > 45 years respond better to a longer Rest-ZZZ supplementation period.

SQ and mood were assessed due to the relationship between sleep and emotional well-being [33]. Supporting the improvements in sleep-related QoL, Rest-ZZZ led to an improvement in TMD score, a measure of overall negative mood states. It is reasonable to expect that the significant improvements in sleep-related QoL for Rest-ZZZ corresponded with significant improvements in TMD. Research has shown that dysfunctional sleep has been linked to impaired emotional functioning and dulled responses to every day events in healthy individuals [33]. Average pre-supplementation POMS Iceberg Profiles showed that participants in all groups had the expected normal profile with a peak in Vigor-Activity. This finding was not changed by the 7-day supplementation of any product, demonstrating the health of the studied population and a lack of detrimental effect of study products on mood.

It is interesting that while participants taking Rest-ZZZ reported improved sleep-related QoL, their satisfaction with SQ was reduced. This discrepancy may be explained by the fact that sleep-related QoL measures the effect of sleep on daytime functioning, while the satisfaction with SQ was subjective and influenced by participants’ expectations for a good sleep. The combination of improved sleep-related QoL with lower satisfaction of SQ may suggest that participants can achieve improvements in daytime functioning without getting as much or as good sleep as they expected. The gap between expectation and need has been documented as sleep need misperception, as individuals may expect more or less sleep than is actually needed, with excessive daytime sleepiness or impaired performance reported in both cases [34]. The sleep need misperception suggests that sleep-related QoL may be a more important SQ parameter than satisfaction with SQ in the overall context of SQ. As with SQ parameters, changes in QoL scores were accompanied by high variability among participants and may be the result of small sample size and short supplementation period. Sleep-related QoL assessed the immediate impact on day-to-day functioning while the Quality of Life Questionnaire measured overall QoL on a more diverse scale. It is possible that the short duration of this study did not provide sufficient time for improvements in sleep-related QoL to translate to improvements in overall QoL, particularly in a healthy population.

The AEs reported while participants were taking Rest-ZZZ suggests that in addition to the potential benefits of more restful sleep and improvements in sleep-related QoL, supplementing with Rest-ZZZ was well tolerated in the studied population. Future investigations into the long-term effectiveness of Rest-ZZZ are unlikely to reveal AEs based on the favorable safety profile established in this study and the natural ingredients in the formulation.

The strengths of this study include the double-blind, randomized, cross-over design and the extensive evaluation of SQ parameters and clinical safety. The HPSQI was designed to provide an assessment of SQ in healthy adults, which included a comprehensive measure of daytime functioning through the assessment of sleep-related QoL. The PSQI [35] is the most popular SQ questionnaire in literature but falls short on providing an assessment of how an individual’s SQ impacts them as a person. The HPSQI consists of seven questions dedicated solely to sleep-related QoL and provides a better illustration of the impact of sleep on daytime functioning. The findings must be considered in the context of the study limitations, which include a small sample size and a short 7-day supplementation period. As such, the results from this pilot study should be confirmed in a larger population of adults over a longer supplementation period. Future investigations should consider assessing SQ by pairing the HPSQI with objective measures, such as actigraphy or polysomnography, to provide a comprehensive measure of SQ that includes objective outcomes such as sleep efficiency, and important subjective outcomes on sleep-related quality of life.

The findings from the PP population suggest that Rest-ZZZ is an efficacious sleep supplement. Rest-ZZZ supplementation resulted in more restful sleep that generated corresponding improvements in sleep-related QoL and mood. Furthermore, subgroup analysis of those ≤ 45 years old demonstrated significant within group improvements in sleep duration, sleep debt, and sleep-related QoL. Efficacy and safety data from this trial suggest that Rest-ZZZ may have a role as a sleep supplement in healthy adults with occasional sleeplessness. Proactively improving sleep in all populations should be considered a priority, as the average North American adult may be on a trajectory towards sleep-related disease. [2, 3].
